# Associated of rs895819 with risk of cervical cancer in Chinese women

**DOI:** 10.7150/jca.46648

**Published:** 2020-08-28

**Authors:** Yu Weng, Bubu Wang, Lingyan Zheng

**Affiliations:** 1 Department of Clinical Laboratory, Sir Run Run Shaw Hospital, Zhejiang University School of Medicine, Hangzhou, China; 2 Department of Clinical Laboratory, The Third Affiliated Hospital, Wenzhou Medical University, Hangzhou, China; 3 Department of Oncology, The Third Affiliated Hospital, Zhejiang University School of Medicine, Hangzhou, China

We have read with great interest the case-control study that Common genetic variants in pre-microRNAs are associated with cervical cancer susceptibility in southern Chinese women by Chen et al 1. The authors' genotyped eight pre-microRNA polymorphisms in 290 cervical cancer patients and 445 cancer-free female controls. In an unconditional logistic regression model by adjusting for age, menopause, delivery and abortion, they found a significant association of pre-miR-27a rs895819 polymorphism with decreased cervical cancer risk. In the discussion, the authors compared this finding with that from a previous study by Xiong et al 2, which showed that rs895819 was associated with reduced risk of cervical cancer in southern Chinese women with 103 cervical cancer patients and 417 controls, and addressed that the findings from two studies were consistent. However, the references for genetic variant comparisons were different between two studies, although both studies were comparable based on the southern Chinese women. We would like to suggest a few improvements to data explanation in the article.

In the study by Chen et al 1, the pre-miR-27a rs895819 T > C polymorphism was shown to significantly decrease cervical cancer susceptibility in dominant model (TC/CC versus TT: odds ratio (OR) = 0.65, confidence interval (CI) = 0.44-0.96, P=0.030), but not in recessive or heterozygous model. In the study by Xiong et al 2, however, the unconditional logistic regression analysis revealed that the risk of cervical cancer was significantly decreased in both homozygotes (TT versus CC: OR = 0.33, 95% CI = 0.15-0.72, P = 0.006) and heterozygotes (TC versus CC: OR = 0.33, 95% CI = 0.15-0.74, P = 0.007), compared with CC homozygotes. Furthermore, a similar trend of the decreased risk of cervical cancer was detected in recessive model (TC/TT versus CC: OR = 0.33, 95% CI = 0.16-0.70, P = 0.004) but not in dominant model. Although both studies found protective effects of rs895819 on cervical cancer, but the references for comparison were different in two studies, i.e., TT as reference in study by Chen et al 1, while CC as reference in study by Xiong et al 2.

We pooled these two studies and re-estimated the effects of rs895819 on the cervical cancer risk by recalculating the crude OR and 95% CI using either TT or CC as reference 3, and found no association between rs895819 and cervical risk except that in a recessive model (Table 1). The results also indicated differential effects of rs895819 on cervical cancer risk between two studies, and in both homogenous and additive models, the effects were controversial when significant associations were found in both studies (Data on forest plots not shown). Thus, the findings were inconsistent between these two studies. These minor explanations could significantly improve completeness of the article and help others for future studies regarding this topic.

It should be noted that both studies performed stratified analyses. The study by Chen et al 1, showed that the pre-miR-27a rs895819 T > C polymorphisms significantly reduced the risk of cervical cancer susceptibility in patients younger than 49 years, those who experienced fewer abortions, and clinical stage I patients. In the study by Xiong et al 2, a more pronounced reduction in cervical cancer risk was observed among clinical stage I patients and squamous cell carcinoma patients carrying rs895819 CT or TT genotypes when comparing with CC genotypes. We did not carry out pooled analyses based on stratified population due to limited data available. It has been reported that rs895819 plays protective role in breast cancer 4 and ovarian cancer 5 among certain group patients. These studies indicate that rs895819 may play protective roles among specific women with female cancers. Future studies are warranted to elucidate effects of rs895819 on cervical cancer among specific patients.

## Authors' contributions

YW conceived of this letter and led the development of the letter to the editor. BW wrote the first draft of the letter, and coordinated and integrated comments, YW, and LZ critically revised and edited successive drafts of the manuscript. All authors read and approved the final version of the manuscript.

## Figures and Tables

**Table 1:** Synthesis analysis of the association between rs895819 and cervical cancer risk

Quantitative synthesis	n^ a^	Heterogenous		Homogenous		Dominant		Recessive		Additive	
		OR (95% CI)^ b^	*P*^ c^	OR (95% CI)^ b^	*P*^ c^	OR (95% CI)^ b^	*P* ^c^	OR (95% CI)^ b^	*P* ^c^	OR (95% CI)^b^	*P*^ c^
Analysis 1^d^	2	0.79 (0.44-1.42)	0.039	1.13 (0.18-7.26)	<0.001	0.86 (0.38-1.93)	0.002	0.47 (0.27-0.82)	0.294	0.94 (0.40-2.19)	<0.001
Analysis 2^e^	2	0.71 (0.20-2.52)	0.015	0.88 (0.14-5.65)	<0.001	0.83 (0.16-4.32)	0.001	1.17 (0.52-2.63)	0.002	1.06 (0.46-2.48)	<0.001

^a^ Number of comparisons. ^b^ The crude OR and 95% CI were calculated based on the genotype frequencies. ^c^ *P* value of Q-test for heterogeneity analysis. ^d^ The genetic variant comparisons in Analysis 1 were TC versus TT, CC versus TT, TC/CC versus TT, CC versus TT/TC and C versus T for heterogenous, homogenous, dominant, recessive and additive model respectively. ^e^ The genetic variant comparisons in Analysis 2 were TC versus CC, TT versus CC, TC/TT versus CC, TT versus CC/TC and T versus C for heterogenous, homogenous, dominant, recessive and additive model respectively.
